# Assessment of 16S rRNA Gene-Based Phylogenetic Diversity of Archaeal Communities in Halite-Crystal Salts Processed from Natural Saharan Saline Systems of Southern Tunisia

**DOI:** 10.3390/biology10050397

**Published:** 2021-05-04

**Authors:** Afef Najjari, Panagiota Stathopoulou, Khaled Elmnasri, Faten Hasnaoui, Ines Zidi, Haitham Sghaier, Hadda Imene Ouzari, Ameur Cherif, George Tsiamis

**Affiliations:** 1 Faculté des Sciences de Tunis, LR03ES03 Laboratoire de Microbiologie et Biomolécules Actives, Université Tunis El Manar, 2092 Tunis, Tunisia; faten.hasnaoui@etudiant-issbat.utm.tn (F.H.); ines.zidi@istmt.utm.tn (I.Z.); imene.ouzari@fst.utm.tn (H.I.O.); 2 Department of Environmental Engineering, Laboratory of Systems Microbiology and Applied Genomics, University of Patras, 2 Seferi Str., 30100 Agrinio, Greece; panstath@upatras.gr (P.S.); gtsiamis@upatras.gr (G.T.); 3 Higher Institute for Biotechnology, University Manouba, BVBGR-LR11ES31, Biotechpole Sidi Thabet, 2020 Ariana, Tunisia; khaled.elmnasri@fst.utm.tn (K.E.); haitham.sghaier@cnstn.rnrt.tn (H.S.); ameur.cherif@uma.tn (A.C.); 4 Laboratory “Energy and Matter for Development of Nuclear Sciences” (LR16CNSTN02), National Center for Nuclear Sciences and Technology (CNSTN), 2020 Sidi Thabet, Tunisia

**Keywords:** archaea, culture approach, DGGE, metagenomic, 16S rRNA gene libraries

## Abstract

**Simple Summary:**

In the current investigation, we determine the archaeal diversity from halite-crystal salts sampled from Chott Djerid and Chott Douz, two saline Chotts in the Tunisian Sahara, using both culture-dependent and independent approaches citing DGGE (V3 regions), 16S rRNA-gene (full length gene ~ 1500 bp) based clone libraries and high-throughput sequencing technology of Illumina MiSeq platform (V3–V4 regions). The amalgamation of all results yielded a comprehensive view of the archaeal diversity represented by members of *Halobacteria* and *Nanohaloarchaea* classes.

**Abstract:**

A thorough assessment of the phylogenetic diversity and community structure of halophilic archaea from three halite-crystal salts, processed from two separated saline systems of Southern Tunisia has been performed using culture dependent and independent methods targeting different regions of 16S rRNA gene sequences including DGGE, 16S rRNA clone libraries and Illumina Miseq sequencing. Two samples, CDR (red halite-crystal salts) and CDW (white halite-crystal salts), were collected from Chott-Eljerid and one sample CDZ (white halite-crystal salts) from Chott Douz. Fourteen isolates were identified as *Halorubrum*, *Haloferax*, *Haloarcula*, and *Halogeometricum* genera members. Culture-independent approach revealed a high diversity of archaeal members present in all samples, represented by the Euryarchaeal phylum and the dominance of the Halobacteria class. *Nanohaloarchaea* were also identified only in white halite samples based on metagenomic analysis. In fact, a total of 61 genera were identified with members of the *Halorhabdus*, *Halonotius*, *Halorubrum*, *Haloarcula*, and unclassified. *Halobacteriaceae* were shared among all samples. Unexpected diversity profiles between samples was observed where the red halite crust sample was considered as the most diverse one. The highest diversity was observed with Miseq approach, nevertheless, some genera were detected only with 16S rRNA clone libraries and cultured approaches.

## 1. Introduction

Hyper-saline lakes are widespread around the world and occur mainly in warm and arid areas where rainwater has been collected, evaporated, and left large deposits of salts (halite, gypsum) and water with a high salt concentration [1]. They are devoid of any kinds of vegetation except some clumps of grass. These areas have been the subject of many microbiological studies for several years and it has been demonstrated that the diversity of halophilic and halotolerant species in such environments is limited, including taxa representing all three domains of life (Archaea, Bacteria, Eukarya) [2,3,4,5,6,7,8,9]. However, the most frequent of these microorganisms are members of the third domain of life, Archaea, especially classified within the family *Halobacteriaceae*, order *Halobacteriales* [10]. They are the main dominant microbial population, in saline systems where NaCl concentration varies from 20% (*w*/*v*) to halite saturation [8,9,11,12,13,14,15,16,17]. These microorganisms (*Haloarchaea*) have been isolated from a wide range of hypersaline ecosystems such as the Dead Sea, the Great Salt Lake (Magna, UT, USA), alkaline brines of Wadi Ntarun (west of the Nile Delta, Egypt), Solar saltern of Sfax Sabkhas (Sfax, Tunisia), Algerian sabkhas (Algeria), Greek solar saltern (Messolonghi, Greece), lake Magadi (Kenya), and saline soils [2,4,7,18,19,20,21,22,23,24]. *Haloarchaea* are known to promote the crystal halite formation [25]. In more detail, when NaCl begins to precipitate, *Haloarchaea* are embedded in situ within fluid inclusions and remain viable for thousands to hundreds of millions of years [26,27,28,29,30]. Therefore, *Haloarchaea* are considered as attractive models for astrobiologists due to their polyextremophilic nature with tolerance of saturating salinity, anaerobic conditions, high levels of ultraviolet and ionizing radiation, subzero temperatures, desiccation, and toxic ions [16,31,32].

Members of *Haloarchaea* lead to an aerobic chemo-organotrophic lifestyle and constitute a physiologically and morphologically distinct group. They are strongly related to high salt concentrations and require a minimum of 1.5 M NaCl concentration for maintaining growth and structural integrity of their cells [4,6,10,25,33,34]. The majority of the *Haloarchaea*—such as *Halobacterium*, *Halorubrum*, *Haloarcula*, *Haloferax*, *Halococcus*, *Halobaculum*, and *Natrialba* spp.—exhibit an optimal growth at near neutral pH [20,35,36,37]. Alkaline saline environments harbor alkaliphilic halobacteria, such as *Natronomonas* and *Natronobacterium* spp., that require at least a pH 8.5 for growth [10,38]. Within *Halobacteriales* at least 51 currently genera have been validly characterized, reflecting their considerable ecophysiological diversity [39,40].

During the last years research has been focused on the study of prokaryotic diversity and the structure of communities in different saline ecosystems from different geographical locations, based on the analyses of PCR-amplified 16S rRNA gene sequencing from environmental samples and using techniques such as DGGE (Denaturing Gradient Gel Electrophoresis), 16S rRNA libraries, SSCP (Single Strand Confirmation Polymorphism), PhyloChip, and 16S rRNA amplicon sequencing [7,20,21,41,42,43]. These approaches have been proved to be powerful tools to assess the microbial diversity since they enable the characterization of novel bacterial and archaeal lineages in such environments [6,7,9,21]. Previous studies on the microbiology of saline environments, indicated that *Haloarchaea* constitute the main dominant microbial group but most of them remain uncultured [21,44,45]. A differentiation of *Haloarchaea* within the different ecosystem studies was also observed [42,45,46,47,48].

Southern Tunisia includes numerous ephemeral inland and costal Saharan salt lakes, known as Sebkhas and Chotts, lying south of the Atlas Mountains at the northern edge of the Sahara [1,2]. These salt ecosystems have been the subject of microbiological studies carried on saline water, sediments, and soils [3,4,5,6]. However, to the best of our knowledge, no studies have unraveled the phylogenetic diversity of archaeal communities of halite-crystal salts from Tunisian ecosystems based on culture dependent and culture independent approaches simultaneously.

The purpose of this study was to assess the archaeal diversity from halite-crystal salts sampled from Chott Djerid and Chott Douz; two saline Chotts in the Tunisian Sahara, using culture dependent and molecular approaches such as DGGE, 16S rRNA-gene based clone libraries and high-throughput sequencing technology of Illumina MiSeq platform. The present study will provide insights of the archaeal diversity from the Tunisian saline ecosystems and may be the first step towards the biotechnological exploitation of the Tunisian archaeal diversity.

## 2. Material and Methods

### 2.1. Site Description and Sampling

**Figure 1:**
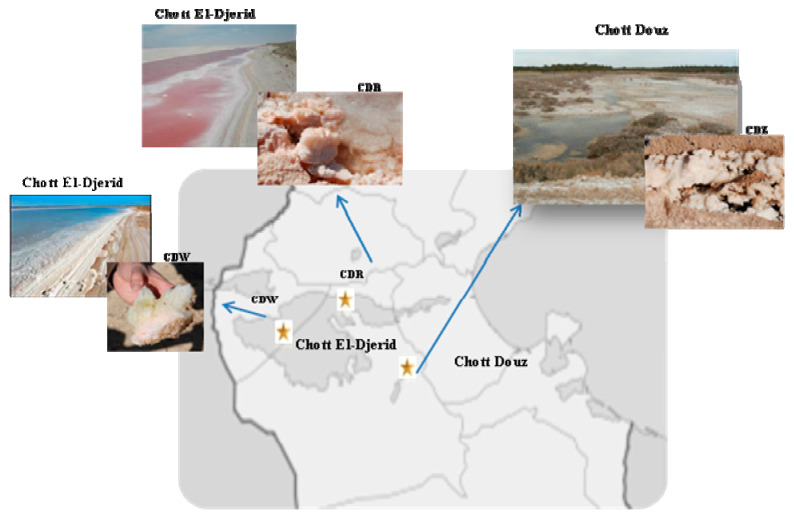
Site map of the different sampling stations. Station 1: Chott El-Djerid (CDR) (N 33°58′736″, E 08°20′632″, Alt 48 ft/14.6 m); Station 2: Chott El-Djerid (CDW) (N 33°57′252″, E 08°24′507″, Alt 7 ft/2 m); Station 3: Chott Douz (CDZ) (N 33°26′753″, E 09°00′814″, Alt 173 ft/53 m).

Samples studied in the present report were collected in February 2010 from arid saline ecosystems located in three different locations including:

(i) Chott Djerid (also known Chott El Djerid), situated in South-West of Tunisia, and it is considered as the largest saline lake (5000 km^2^) of the Sahara with a surface area of over 5000 km^2^. Chott Djerid is a seasonal lake, that is completely dry for most of the year. It represents an important source of NaCl production for Tunisia. Two representative halite-crystal salts (white and red pigmentation), were collected from two different sites named as BDV18.2 (CDR) (GPS: N 33°55′10″ E 8°30′8″ Al 16 m) and BDV19.2 (CDW) (N 33°55′55″ E 8°28′19″ Al 9 m) respectively (Figure 1).

(ii) Chott Douz, located in the South of Tunisia at 125 km South-East of Chott Djerid (GPS N 33°26,845′ E 9°1356′ Al 64 m). White halite-crystal salts sample indicated as CDZ was used in this study (Figure 1).

Generally, the pH of the two Chotts was quite close to the neutrality (~7.4). The surrounding environment was characterized by the extremely low relative humidity. The temperature was ranging from 4 °C in the night and 38 to 40 °C during the day. All samples were collected under aseptic conditions into 50-mL sterile falcon tubes and were kept at 4 °C until further processing.

### 2.2. DNA Extraction from Environmental Samples and Archaeal Isolates

Total DNA from salt samples was extracted using the FastDNA spin kit for Soil (MoBio, Carlsbad, CA, USA) according to the manufacture procedure. Quantification of nucleic acid samples was performed with a Thermo Scientific NanoDrop^™^ ND-2000 Spectrophotometer (Thermo Fisher Scientific, Waltham, MA, USA) according to the manufacturer’s instructions. Genomic DNA from isolates was extracted from log-phase cells, lysed in distilled water by phenol-chloroform method and subsequently precipitated with ethanol, as previously described [49].

### 2.3. Isolation of Halophilic Archaea, PCR Amplification, and 16S rRNA Gene Sequencing

Enrichment and isolation of aerobic extreme halophiles were performed on DSC-97 medium [47] consisting of: casamino acids, 7.0 g, yeast extract, 10.0 g, trisodium citrate, 3.0 g, KCl, 2.0 g, MgSO4.7H_2_O, 20.0 g, FeCl_2_, 0.023 g, NaNO_3_g, 0.04 g, MnCl_2_, 0.025 g, NaBr, 0.5 g, NaCl, 250 g, Agar-agar, 20 g, distilled water, 1000 mL, pH 7.4. One gram of each salt sample was added to 25 mL of medium in 100 mL Erlenmeyer flasks in a rotary shaker at 37 °C under agitation 120 rpm for 5 days. Serial dilutions of enriched cells were then plated onto agar medium. The plates were incubated for four weeks at 30 °C. Colonies were then selected based on their morphologies (size, margin, color). Cells were maintained as glycerol stocks (25%, *v*/*v*) at—80 °C. The ability of haloarchaeal isolates to grow in different temperatures (4, 20, 30, 35, 40, 45, and 50 °C), pH (4–9) and in various salt concentrations (0–30%), was evaluated.

Molecular identification was based on 16S rRNA gene amplification and sequencing using universal archaeal primers 4F (5′-TCCGGTTGATCCTGCRG-3′) and 1492R (5′-GGTTACCTTGTTACGACTT3-′) [50]. PCR reactions were performed in 50 µL reaction mixtures containing the following: PCR buffer (1X), MgCl_2_ (1.5 mM), 0.25 mM of each dNTP, 0.5 µM of each primer, 1 µg of chromosomal DNA, and 1 U of Taq DNA polymerase (Fementas). The program used was: 95 °C for 5 min; 35 cycles of 94 °C 45 s, 64 °C 45 s, 72 °C 1 min, and a final extension step at 72 °C for 10 min. The presence of specific PCR product was visualized after electrophoresis on 1.5% (*w*/*v*) agarose gels under UV light. PCR products were then purified using the polyethylene glycol (PEG) precipitation protocol [51]. Sequencing was performed in both strands on an ABI3130 analyzer according to the manufacturer’s instructions (Applied Biosystems, Waltham, MA, USA).

### 2.4. DGGE Fingerprinting

To assess the total archaeal community structures among the salt samples, DGGE fingerprinting approach was applied. A nested PCR technique was employed to amplify the variable V3 region of 16S rRNA gene sequences from archaea as previously described by Cindy et al. (2001) with slight modifications. In the first round of PCR two primers, PRA46F (5′-YTAAGCCATGCRAGT-3′) and PREA1100R (5′-YGGGTCTCGCTCGTTRCC-3′), were used in order to amplify the archaeal community [52]. PCR reactions were carried out in a final volume of 20 µL contained buffer 1 X (Fermentas), MgCl_2_ 1.5 mM, 0.25 mM of each dNTP, 0.25 µM of each primer, 1–5 ng of total DNA, and 1 U of Taq DNA polymerase (Fementas). The program used was: 95 °C for 5 min; 35 cycles of 94 °C 45 s, 43 °C 45 s, 72 °C 1 min, and a final extension step at 72 °C for 10 min. Five to 10 µl of the previously PCR product were used as a template for the second PCR under the same conditions using PARCH340F-GC (5′-CCCTAYGGGGYGCASCAG3′) and PARCH 907R (5′-GWATTACCGCGGCKGCTG-3′) as primers [53]. A 40 bp of GC clamp (CGCCCGCCGCGCGCGGCGGGCGGGGCGGGGGCACGGGGGG) was added to primer PARCH340F to increase separation of DNA bands in DGGE analysis [54].

Profiles of the amplified 16S rRNA gene sequences were produced by DGGE as described by Muyzer et al. [54] with some modifications. One hundred ng of the PCR products were loaded onto 8% (*m*/*v*) polyacrylamide gel in 0.5X TAE buffer (20 mM Tris (pH 8.0), 10 mM acetic acid, 0.5 mM EDTA; Bio-Rad, Paris, France) with a gradient between 5 and 55% (100% denaturant contains 7 M urea and 40% (vol/vol) deionized formamide). The electrophoresis was carried out in 0.5X TAE buffer at 200 V for 5 h at a constant temperature of 60 °C. The DNA fragments were stained for 30 min in 0.5X TAE buffer with ethidium bromide and visualized under UV light. The gels were then washed in distilled water for 15 min. DNA bands from the polyacrylamide gels were excised and incubated in 80 μL of sterile distilled water overnight at 37 °C. Five to 10 μL portion of the eluted DNA was used as the template in a PCR re-amplification with the PARCH 340F (without GC clamp) and PARCH 907R. PCR products were verified on 2% (*w*/*v*) agarose gel, purified with QIAquick PCR Purification Kit (Qiagen, Hilden, Germany) and sequenced by PRIMM (Milano, Italy). Sequencing was performed in one sense with primer PARCH340F.

### 2.5. Full-Length 16S rRNA Archaeal Libraries

Amplification of nearly complete 16S rRNA gene sequences from total environmental DNA of salt samples was performed using universal primers for archaea: 4F (5′-TCCGGTTGATCCTGCRG-3′) and 1492R (5′-GGTTACCTTGTTACGACTT3-’) [50,55]. PCR reactions were carried out in a final volume of 50 μL containing: 1X buffer (Takara), 1.5 mm MgCl_2_, 0.25 mM of each deoxynucleoside triphosphate, 0.3 mM of each primer, and 1 U Taq polymerase (Takara). PCR reactions were performed using a PTC-200 thermocycler (MJ Research Inc., Reno, NV, USA) with the following program 94 °C for 10 min; 35 cycles of [94 °C 45 s, 53 °C 45 s, 72 °C 1 min], and a final extension step at 72 °C for 10 min. PCR products verified on 1.2% (*w*/*v*) agarose gel electrophoresis and purified by PEG precipitation [56]. Purified DNA was cloned into pGEM-T, according to the manufacturer’s protocol (Promega, Madison, WI, USA), and transformed into competent *Escherichia coli* DH5α cells. White colonies on ampicillin/X-gal plates were screened for inserts of the correct length by PCR with the pGEM-T compatible primers T7 and SP6. Inserts were sequenced using an ABI3130 analyzer according to the manufacturer’s instructions (Applied Biosystems, Waltham, MA, USA).

### 2.6. Sequence Analysis and Phylogenetic Assessment of Archaeal 16SrRNA Gene Sequences of Isolates and Clone Libraries

The nearly complete 16S rRNA gene sequences were assembled using DNAstar software (DNASTAR Inc., Madison, WI, USA). Sequence similarity searches were performed using the ‘‘Seqmatch’’ (Ribosomal Database Project II (http://rdp.cme.msu.edu/seqmatch/seqmatch_intro.jsp, accessed on 10 December 2020) against sequences from individual isolates (type strains) and environmental uncultured sequences. For each isolate and clone sequences the two nearest relatives were obtained for phylogenetic analyses. The one with the highest percentage of similarity was retained for homology.

For the 16S rRNA archaeal libraries clones were initially screened for possible chimeric structures using the program CHECK_CHIMERA of the Ribosomal Database Project (RDP-II) [57]. After sequencing, clustering analysis was carried out using the Mothur package at the 3% cutoff, and the nearest relative was assigned using the Ribosomal Database Project [57,58]. Species richness (alpha diversity) was evaluated based on the diversity indices values notably the Chao1 estimator, Shannon diversity index, and Good’s coverage calculated with the Mothur software version 1.28.0 [58].

The alignment of sequences was carried out using the CLUSTALX 1.83 program. The distance matrix-based phylogenetic tree was created using the PAUP* 4b10 software package. The distances were calculated using the Jukes and Cantor method [59] and the Topology was inferred using the “neighbor-joining” method [60] based on a bootstrap analysis of 1000 trees. The maximum parsimony calculated phylogenetic tree using the PAUP phylogenetic package was also generated. Only sequences longer than 200 bp were used in tree construction.

### 2.7. Illumina MiSeq Sequencing, Data Analysis, and Diversity Estimates

As an additional technique, the archaeal diversity was performed by next generation sequencing the 16S rRNA gene, using the Illumina MiSeq platform. Polymerase chain reaction (PCR) was performed with KAPA HiFi HotStart ReadyMix PCR Kit (KAPA BioSystems, Cape Town, CA, USA) and extracted DNA as a template. Targeted variable regions (V3–V4) of the archaeal 16S rRNA gene was amplified with the primer pair Arc340F-MiSeq 5′-TCG-TCG-GCA-GCG-TCA-GAT-GTG-TAT-AAG-AGA-CAG-CCC-TAC-GGG-GYG-CAS-CAG-3′ and Arc806R-MiSeq 5′-GTC-TCG-TGG-GCT-CGG-AGA-TGT-GTA-TAA-GAG-ACA-GGG-ACT-ACV-SGG-GTA-TCT-AAT-3′. The amplification reaction mixture consisted of 1X reaction buffer, 0.28 mM of dNTPs, 0.28 mM of each primer solution, 0.012 U of KAPA HiFi HotStart DNA Polymerase solution and 1 μL from the template DNA solution. The PCR conditions were as follows: 3 min denaturation at 95 °C; 30 cycles of 98 °C for 20 s, 60 °C for 15 s and 72 °C for 45 s, and 1 min of final elongation step at 72 °C. All PCR products were migrated on a 1.5% (*w*/*v*) agarose gel in 1X Tris-Acetate-EDTA buffer (40 mM Tris–acetate, 1 mM EDTA). The amplified 16S rRNA products were purified with a 20% PEG, 2.5 M NaCl solution. The concentration of purified DNA was measured with a Quawell Q5000 micro-volume UV–vis spectrophotometer.

The resulting PCR amplicons were first diluted up to 10 ng/μL and then used for a second-step PCR to include the indexes (barcodes) as well as the Illumina adaptors. The combinatorial use of index primers resulted in unique samples that were pooled and sequenced on one Illumina MiSeq run. Amplification reaction was performed in a final volume of 50 μL contained 1X of KAPA HiFi Fidelity Buffer, 0.3 mM dNTPs, 1 μM of the forward and the reverse indexing primers, 0.02 U of KAPA HiFi HotStart DNA Polymerase, 2 μL from the diluted PCR product (10 ng/μL) and 25.5 μL of sterile deionized water. The program of PCR amplifications consists of 3 min incubation at 95 °C followed by 8 cycles of 95 °C for 30 s, 55 °C for 30 s and 72 °C for 30 s, and a final elongation step at 72 °C for 5 min. The resulting amplicons were purified using Macherey-Nagel’s NucleoMag^®^ NGS Clean-up and Size Selection kit according to the manufacturer’s recommendations, and all amplicons were quantified with a Quawell Q5000 micro-volume UV–vis spectrophotometer and merged in equimolar ratios (8 nM). The paired-end (2 × 300 bp) sequencing was done by Macrogen (Seoul, Korea) on the Illumina MiSeq platform.

Analysis of sequenced reads was done according to the standard pipeline of DADA2 (Divisive Amplicon Denoising Algorithm2) following the related standard operating procedure pipeline [61]. In brief, sequences that contained one or more ambiguous bases (N), sequences having a homopolymer stretch longer than 7 bases, and sequences shorter than 200 were considered of poor quality and trimmed from the data set. Chimeras were removed as well. Then, high-quality reads were aligned against the SILVA alignment database version silva_nr_v128 was applied. Filtered alignments were selected to generate a pairwise distance matrix, followed by binning the sequences into operational taxonomic units (OTUs). The mothur software package was applied to compute alpha diversity including richness, evenness, diversity and Good’s coverage indices. Indeed, rarefaction curves-based ranking were calculated using resampling-without-replacement approach [58].

## 3. Nucleotide Sequence Accession Numbers

All 16S rRNA sequences identified in this study were deposited in GenBank with the following accession numbers: MW565874-MW565887 for isolates; MW564198-MW564204 for DGGE; MW564054-MW564064 for CDZ sample; MW563869-MW563906 for CDW; and MW565874-MW565920 for CDR sample.

## 4. Results and Discussion

### 4.1. Isolation and Identification of Haloarchaeal Isolates

**Figure 2:**
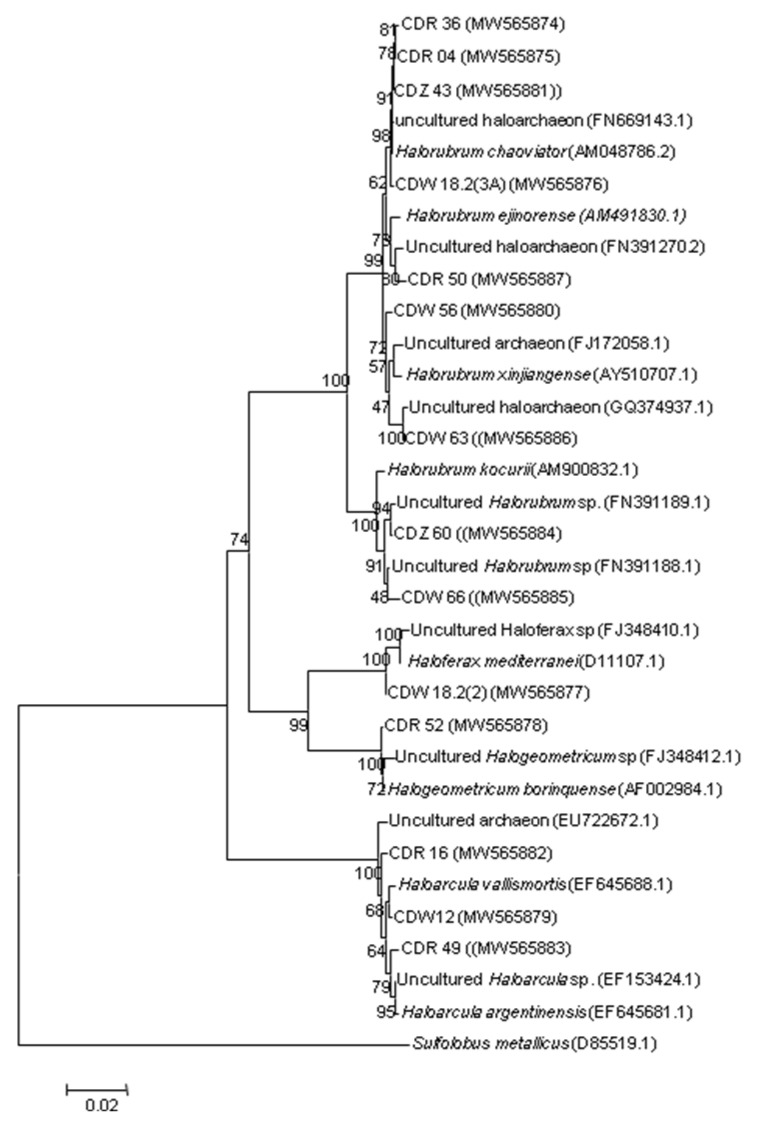
Phylogenetic relationships among haloarchaeal group members, isolated from halite-crystal salts (CDR, CDW, and CDZ), and their closest relatives retrieved from RDPII database. Evolutionary distances were calculated using the method of Maximum Composite Likelihood and the topology was inferred using the neighbor-joining method using MEGA 6. Numbers on the nodes present % bootstrap values based on 1000 replicates. Scale bar represents 0.02 substitutions per site. The 16S rRNA gene sequence of *Sulfolobus metallicus* was arbitrarily chosen as the outgroup to define the root of the tree. Accession numbers of sequences are shown in parenthesis.

Our culture-dependent approach led to the isolation of 44 archaeal isolates that were grouped into 14 categories based on their morphology and color. All groups were then subjected to phenotypical and molecular characterization (Figure 1 and Table 1). Almost all isolates produced red, pink or orange pigmentation on agar plates which may be related with the presence of carotenoids in their cell membranes or with the C50 compound bacterioruberin and its derivatives. These pigments are known to protect haloarchaeal cells from extremophilic conditions such as osmotic stress, damage from high ultraviolet light exposure and anoxic or anaerobic conditions [62,63]. They contribute also in the salt crystallization process [63,64]. Bacterioruberin pigment content in some saline ecosystems has been used to survey the haloarchaeal community in such ecosystems [65]. White colonies were also isolated from CDW halite-crystal salt sample, similar to those were described in another study regarding the isolation of halophilic archaea from salt samples [66]. All observed colonies were circular, convex, mucoid, or not, with entire edges (Table 1). Their cells were pleomorphic or rod-shaped and stained Gram-negative as expected. All isolates were extremely halophilic and required between 10–30% of NaCl for growth with an optimal at 25% NaCl (*w*/*v*) (Table 1). Isolates also showed a large range of growth temperature from 4 °C to 45 °C with an optimal growth at 40 °C; and pH ranging from 6 to 9 with the an optimum at 7.4 (Table 1). Hence, based on phenotypic characteristics, isolates were presumably assigned to the *Halobacteriacaea* family [10,11,13,33]. In order, to confirm this assignment, the 16S rRNA genes of all isolates were sequenced and were then compared to sequences deposited in the RDP database including cultured and uncultured strains (Table 1). The use of both distance- matrix and character-based (parsimony) methods enabled the construction of related phylogenetic trees. All isolates were placed within the family *Halobacteriaceae* and assigned to the following taxonomically described species with a similarity of 99%: *Halorubrum chaoviator* (AM048786) (*n* = 4), *Halorubrum xinjiangense* (AY510707) (*n* = 1), *Haloferax mediterranei* (D11107) (*n* = 1), *Haloarcula vallismortis* (EF645688) (*n* = 2), *Halogeometricum borinquense* (AF002984) (*n* = 1), uncultured *Haloarcula* sp. (EF153424) (*n* = 1), uncultured haloarchaeon (FN391270; GQ374937) (*n* = 2), and uncultured *Halorubrum* sp. (FN391188) (*n* = 2) (Table 1 and Figure 2).

The 16S rRNA gene sequences similarity percentages and phylogenetic affiliations are shown in Table 1, and all of them shared 99% similarity with their closest phylogenetic relative. Our results are in accordance with those reported the diversity of isolated halophilic archaea from salt samples—such as rock salt, halite, gypsum, ancient halite, and salt brines—where isolates were mainly affiliated to the class *Halobacteria* (phylum *Euryarchaeota*) and particularly within the genera *Haloarcula* and *Halorubrum*, while representatives from other genera are less common [30,67,68,69,70,71,72,73]. These extremely halophilic archaea are known to remain viable inside halite and salt brine for months and years [27,30,74].

### 4.2. Total Archaeal Community Assessment

#### 4.2.1. DGGE Fingerprint

As a preliminary step to get an overview in the differences in community profiles of archaeal population thriving in halite-crystal salts samples, DGGE analysis was carried out. A denaturing gradient 20–50% was performed (Figure S1).

**Figure 3:**
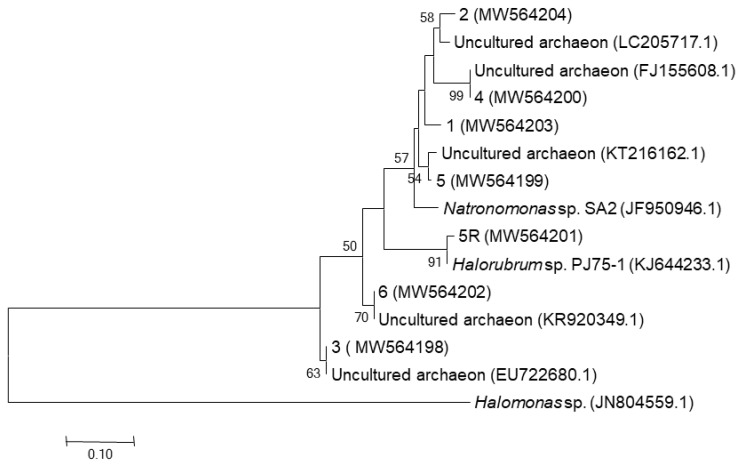
Maximum likelihood phylogenetic tree inferred from 16S rRNA gene sequences of DGGE bands and their closest relatives retrieved from RDPII database. Tree topology was build using the neighbor_joining method using MEGA 6.0. Bootstrap values (*n* = 1000 replicates) were indicated at the nodes. Scale bar represents 5% sequence difference. *Halomonas* sp. (JN804559.1) was used as the outgroup to define the root of the tree. Accession number of sequences are shown in parenthesis.

The resulting DGGE pattern highlighted different bands (*n* = 7) with diverse level of migration and intensity. Each band represents an archaeon-taxon and the presence of two similar migrating bands suggests that the same taxon was present in all samples. It is worth noting that almost all observed bands were encountered in the bottom of the gel reflecting their high % GC content, similar with the profile of *Halobacteriaceae* members, whose GC base ratios are exclusively high and range between 62–68% [75]. Close to these results is the survey of Archaea by Tamez and Lopes-Cortes [76]. CDR and CDW revealed a nearly similar DGGE fingerprint (three bands) and this similarity is rather expected due to the same sampling location (Chott Djerid). Moreover, CDZ revealed a completely different DGGE profile, characterized by the presence of two intense bands in the bottom of the gel, reflecting the low diversity of Archaea in that sample. Seven representative bands from different levels of migration were excised, re-amplified successfully, and finally sequenced. Data obtained indicated that archaeal communities in the three salt samples were affiliated to *Euryarchaeota* phylum (Table 2 and Figure 3). Most of the bands occurred in all samples were assigned to uncultured and unclassified_*Halobacteriaceae* (95.95–100% identity sequences) which is in accordance with previously published data on salt samples analyzed with DGGE technique [77]. Additionally, the other bands were assigned to cultivated *Haloarchaea* such as *Natronomonas* sp. with a 95% of identity observed only in CDZ, and *Halorubrum* sp identified in CDR with a 97.97% sequence similarity.

Comparative analyses of the DGGE sequences and their closely relatives are illustrated in Figure 3. The neighbor-joining phylogenetic tree showed four main clusters corresponding to the phyla *Euryarchaeota*: three clusters, corresponding to order *Halobacteriales*, where the first could be assigned to *Natrinema* group encompassing the bands (2, 1, 4, and 5), the second represented by one band (5R) assigned to *Halorubrum* species and the rest assigned to unclassified uncultured *Halobacteriacaea*. It is worth noting that, limited research on the analysis of haloarchaeal community structure in halite crystals and salt samples, turned out that *Halobacteriales* members constituted the main group [53,78]. It is interesting to note that the distribution of archaeal members observed in our samples could be related to the adaptation of these microorganisms to some extreme physicochemical parameters such as salinity, pH, temperature, and oxygen [79,80,81].

#### 4.2.2. Full Length 16S rRNA Gene Libraries

Archaeal community structure lying in different salt samples was further investigated by cloning and sequencing nearly the complete sequence of 16S rRNA genes from total DNA for the three samples (CDW, CDR and CDZ). All sequence reads (*n* = 90) were compared versus the reference database of known 16S rRNA genes obtained from RDP program. Operational taxonomic units were assigned based on a similarity distance threshold of 0.03 and their specific allocation was: 18, 12 and 2 OTUs in CDR, CDW, and CDZ respectively (Table 3). The results showed that all clones were affiliated to *Euryarchaeaota* phylum with a diverse assemblage of classified and unclassified *Halobacteriales* members (Table 3).

##### Diversity and Richness Estimates

Alpha diversity of archaeal diversity was evaluated based on Shannon index, non-parametric richness (Chao) and Good’s Coverage estimations (GC) (Table 4). The results showed that based on the GC estimates the percentage of the total species were the order of 100%, 95%, and 76.31% for CDZ, CDW, and CDR respectively (Table 4), reflecting a sufficient sequencing depth for CDZ and CDR samples. In terms of archaeal richness, CDZ (25.2) had the highest chao value; followed by CDW (12.16) and CDZ (2.0) had the lowest values.

##### Taxonomic Assignment, Quantification, and Distribution

**Figure 4:**
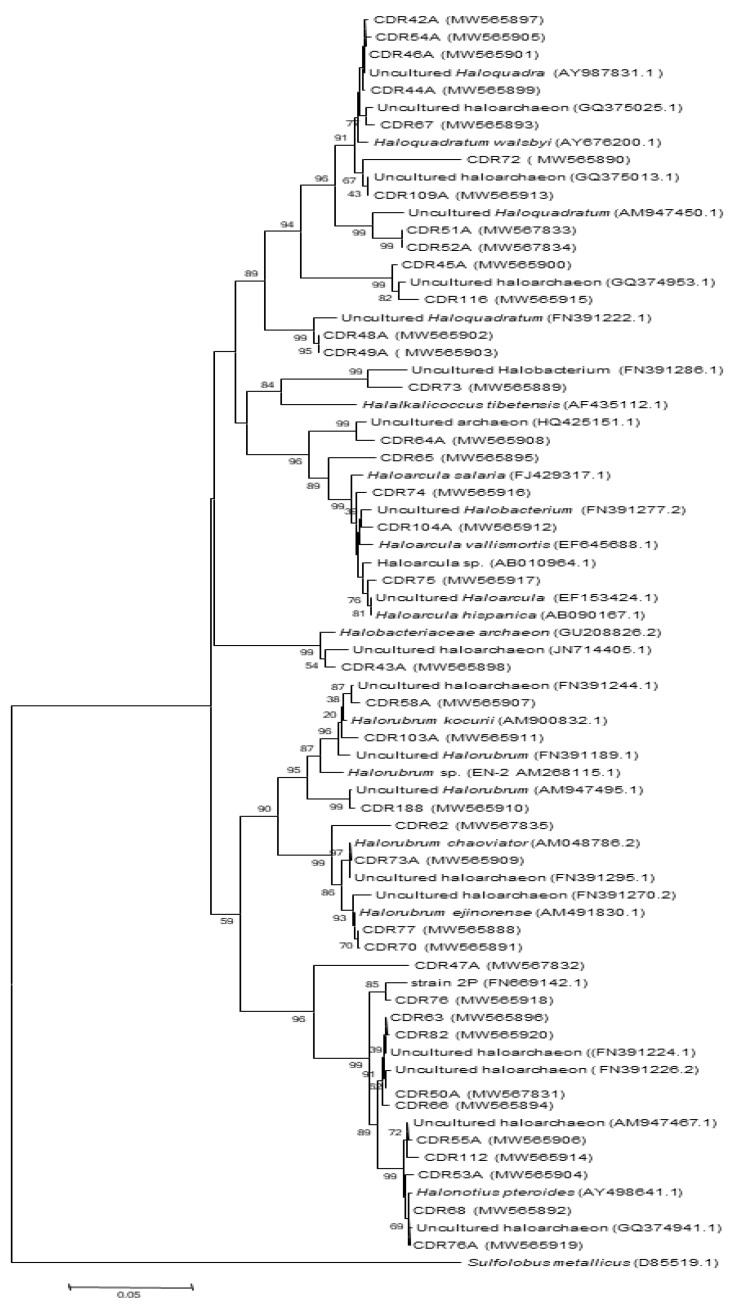
Neighbor-joining tree showing relationships among CDR clones’ and their closest relatives retrieved from RDPII database. Evolutionary distances were calculated using the method of maximum composite likelihood and the topology was inferred using the neighbor-joining method using MEGA 6 software. Numbers on the nodes present % bootstrap values based on 1000 replicates. Scale bar represents 0.05 substitutions per site. The 16S rRNA gene sequence of *Sulfolobus metallicus* was arbitrarily chosen as the outgroup to define the root of the tree. Accession number of sequences are shown in parenthesis.

(i) In CDR sample 38 clones were obtained and were clustered in 18 OTUs. Almost all of them were phylogenetically assigned to uncultured *Halobacteriales* members (Table 3 and Figure 4). The clones were most closely related to 6 genera of extremely halophilic archaea including *Halonotius*-like sequences (OTU9, 10, and 11), *Haloquadratum*-like sequences (OTU1, 2, and 3), *Halorubrum*-like sequences (OTU5, 6, 7, and 8), *Haloarcula*-like sequences (OTU12 and 13), *Halolamina* (OTU4) and *Halorubellus*-like sequences (OTU14). The remaining OTUs (15 to 18) (15.78%) were phylogenetically unrelated to any previously cultivated taxa and are putative candidates for new genus and species-levels members in *Halobacteriaceae* family (Table 3). Members of the identified genera are consistent with that generally observed in many salt-saturated habitats like in several Tunisian hypersaline lakes [7,21,23], in Australian crystallizer ponds [82], in Santa Pola saltern in Spain [83] and in Keke Salt Lake [84]. Generally, such hypersaline habitats were known to hold high densities of extremely halophilic archaea characterized by a restricted number of genera—mainly *Halotonuis*, *Halorubrum*, and *Haloarcula* [9,85,86].

In addition to the above observations, sequences affiliated to *Halonotius*, *Haloquadratum*, and *Halorubrum* genera dominated the clone libraries of CDR sample with a relative abundance of 26.3%, 21%, and 18.4% respectively. Ubiquity and abundance of *Halonotuis* genus members have been observed also in several hypersaline ecosystems such as saline lakes in Australia and China [84,87] and in solar salterns in Turkey and Spain [9,88]. Currently, two species are described, *Halonotius pteroides*, isolated from a crystallizer of an Australian saltern [43,89] and *Halonotius* aquaticus, isolated from a marine saltern in Spain [9]. On the other hand, the *Haloquadratum* genus, characterized by its peculiar square shape, was initially discovered in 1980 [90] and isolated in 2004 by Bolhuis and his colleague [91] and Bolhuis and his collaborators [92]. Similar to *Halonotius*, it was observed in many hypersaline habitats close to saturation such as salt crystals and saturated thalassic lakes [21,93,94]. Concerning the third observed genus, *Halorubrum*, proposed by McGenity [95], is considered as the largest Haloarchaeal genus with 42 valid species [40]. It is a well-known and omnipresent genus within the *Halobacteria*, whose members have been detected specially in salt and halite samples in several extremely saline environments—e.g., salterns, saline lakes, dead sea, salt-saturated crystallizer ponds, and ancient salt deposits [17,46,47,96]. Genetic diversity of, *Halorubrum* may due to high levels of both recombination and horizontal gene transfer [97].

The second subdominant group observed in CDR sample, was represented only by *Haloracula* genus members with a relative abundance of 10.52%. Similarly to *Halorubrum*, *Haloarcula* species are known to reside in the salt-saturated samples [98]. They are able to survive during the fluctuation of salinity and desiccation inside halite crystals. Actually, *Haloarcula* genus encompasses thirteen species [99].

**Figure 5:**
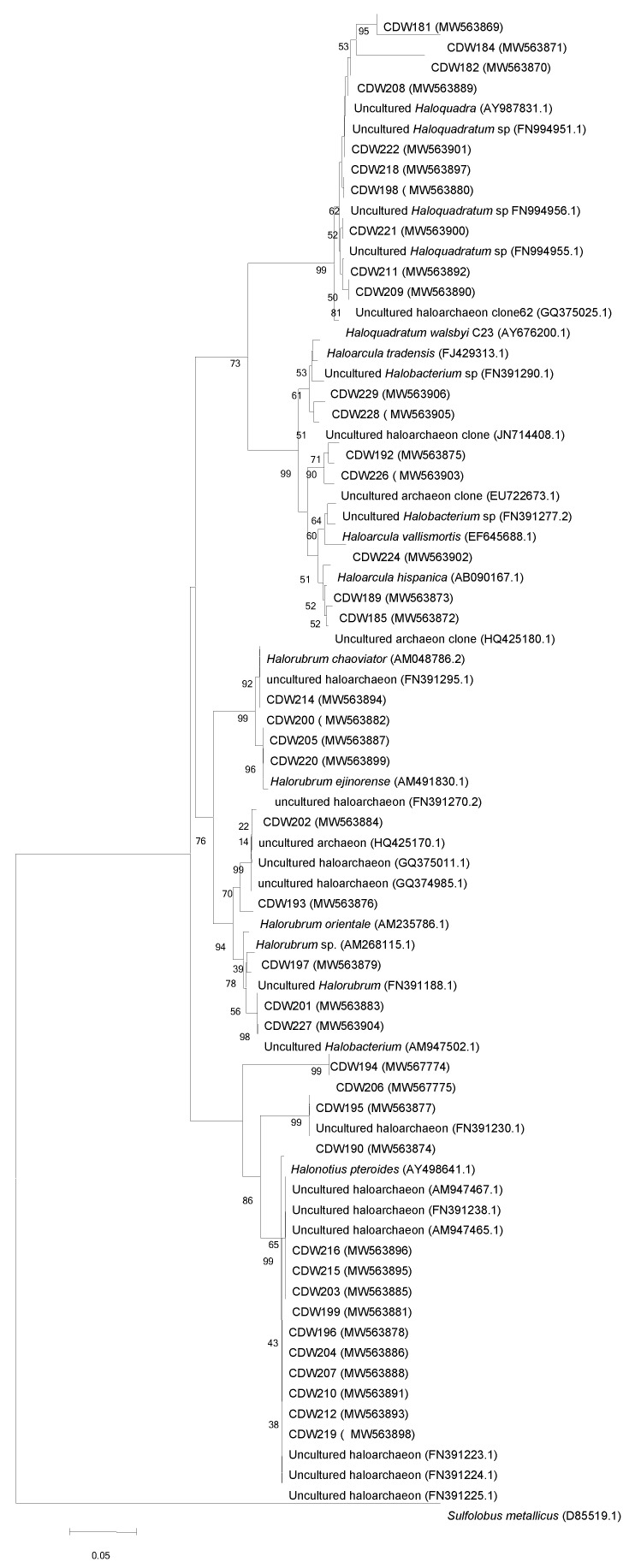
Neighbor-joining tree showing relationships among CDW clones’ and their closest relatives retrieved from RDPII database. Evolutionary distances were calculated using the method of maximum composite likelihood and the topology was inferred using the neighbor-joining method using MEGA 6 software. Numbers on the nodes present % bootstrap values based on 1000 replicates. Scale bar represents 0.05 substitutions per site. The 16S rRNA gene sequence of *Sulfolobus metallicus* was arbitrarily chosen as the outgroup to define the root of the tree. Accession number of sequences are shown in parenthesis.

**Figure 6:**
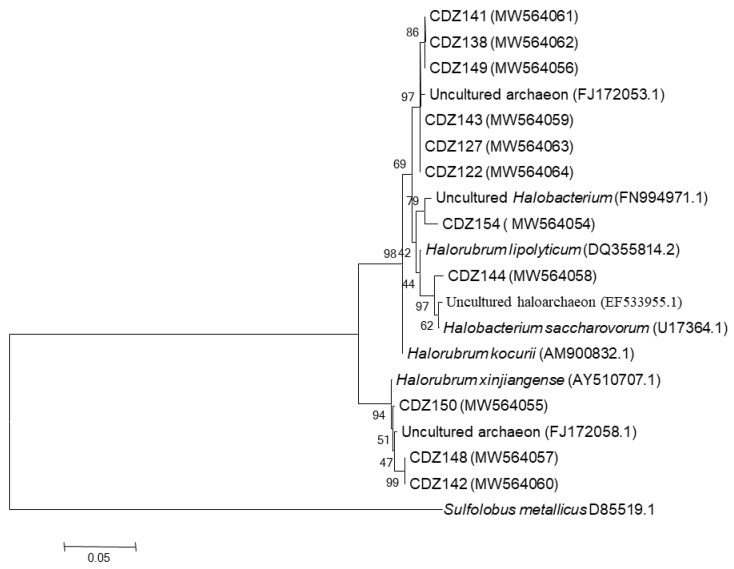
Neighbor-joining tree showing relationships among CDZ clones’ and their closest relatives retrieved from RDPII database. Evolutionary distances were calculated using the method of maximum composite likelihood and the topology was inferred using the neighbor-joining method using MEGA 6 software. Numbers on the nodes present % bootstrap values based on 1000 replicates. Scale bar represents 0.05 substitutions per site. The 16S rRNA gene sequence of *Sulfolobus metallicus* was arbitrarily chosen as the outgroup to define the root of the tree. Accession number of sequences are shown in parenthesis.

The lowest abundant genera found were *Halolamina* and *Halorubellus* each one represented with 2.63% of the total clones. Generally, these two genera were most abundant in environments with fluctuating degrees of salinity [100,101]. The genus *Halolamina* contains only one described species isolated from an artificial marine saltern in China [100] Two species were described for *Halorubellus* genus, *Halorubellus salinus*, and *Halorubellus litoreus* [100,101].

(ii) In CDW sample, a total of 40 clones were obtained and phylogenetically clustered in 12 OTUs unevenly (Table 3 and Figure 5). All OTUs, were identified as uncultured *Halobacteriales* members (Table 3). Four genera, already observed in the CDR sample, were identified including *Halonotius*-like sequences (OTU6 and 7) with relative abundance of 30%, *Haloquadratum*-like sequences (OTU1, 2, and 3), *Halorubrum*-like sequences (OTU8 and 12) and *Haloarcula*-like sequences (OTU9), with a relative abundance of 25%, 22.5%, and 7.5% respectively (Table 3). The remaining *Halobacteriales* clones (OTU10, 11, and 4) with a relative abundance of 12.5% were not affiliated to any described genera and were therefore form a new phylogenetic lineage (Table 3). Similar unclassified *Halobacteriales* members have been described in other extreme saline environments as well [21,44,102,103]. The distribution of the affiliation of clones’ sequences of CDW is more or less similarly to CDR, except for the unclassified *Halobacteriaceae* which they were overrepresented in CDR (33%) than in CDW (12%).

(iii) CDZ sample was characterized by a restricted number of clones (*n* = 11) and whence a low number of OTUs (*n* = 2), and all of them were affiliated to uncultured *Halorubrum* related genus members (Table 3 and Figure 6).

Collectively, these results gave a general idea on the diversity of archaeal community members in three different halite-crystal salts samples based on almost complete 16S rRNA gene libraries. Globally, the community was dominated by clones’ sequence affiliated to *Halotonius*-like sequences genera in the two samples CDR and CDW, and the members of *Halorubrum* genus were observed in the three halite-crystal salts samples. Our results are in accordance with other studies assessing the diversity of halophilic Archaea in saturated salt samples like in Korean (33% salt), Secovlje (25–30% salt) and Maras salterns (25–31% salt) showing that almost all sequences were grouped within members of *Halorubrum* genus [104].

#### 4.2.3. Archaeal Profiling Using MiSeq-Based High-Throughput 16S rRNA Sequencing Approach

A third method was used to improve our understanding on archaeal population present in the three halite-crystal salts based on MiSeq sequencing using the Illumina platform. A total of 95,144 16S rRNA reads for the three datasets was obtained. It is worth noting that only OTUs with a relative abundance ≥1% were considered in the analysis.

##### Diversity and Richness Estimates

An overview on the alpha diversity indices of the archaeal community is illustrated in Table 4. A good coverage rate was obtained at cut-off value of 3% for all samples (Figure S2). Good’s coverage with values >99% displays saturated sampling of all samples which indicate almost sampling completeness (Table 4). Indeed, the results indicated that CDR had the greatest archaeal diversity, followed by CDW and CDZ (Shannon indices of 3.55, 3.25, and 1.245, respectively). In terms of archaeal richness, CDR (134.7) had the greatest chao1 value, and CDZ (11) had the lowest one.

##### Class and Genus Levels Distribution

At class level, all OTUs were assigned to the class *Halobacteria* for CDZ sample, while for CDW and CDR samples archaeal representatives span the classes *Halobacteria* with a relative abundance of 95.73% and 88.95% respectively, and *Nanohaloarchaea* with a relative abundance of 4.62% and 11.04% for CDR and CDW respectively. The dominance of members of *Halobacteria* versus *Nanohaloarchaea* is in agreement with previous reports focused on the study of archaeal diversity in NaCl saturated samples like halite nodules, crystallizer ponds, crusts of halite and commercial salts, based on high- throughput sequencing methods [105,106,107,108,109]. It is interesting that *Nanohaloarchaea* was not reported in earlier studies as a component of archaeal community in hypersaline environment because of limitations of the approaches used before to evaluate the microbial diversity until the introduction of NGS technologies [110], citing the examples of the mismatches of primers used targeting the 16S rRNA gene copies with *Nanohaloarchaea* genes regions’ or the lack of their sequences in databases used for similarity searches [111,112]. In fact, *Nanohaloarchaea*, is a group of nanosized halophilic archaea lineage first recuperated from the soda lake Magadi in the east-African Rift Valley [113] and then from several hypersaline ecosystems where some complete genomes of uncultivated “*Candidatus Nanohaloarchaea*” have been assembled from metagenomes analysis [114,115]. *Nanohaloarchaea* were first affiliated to *Halobacteria* class within the phylum *Euryarchaeota* based on 16S rRNA phylogeny and then based on the shotgun genomes assembling, a novel phylum “*Nanohaloarchaeota*” within a “DPANN” superphylum (*Diapherotrites*, *Parvarchaeota*, *Aenigmarchaeota*, *Nanoarchaeota*, and *Nanohaloarchaea*) was proposed [106,116,117,118]. *Nanohaloarchaea* members are characterized by small size, a one-copy rRNA operon and low ***GC***-content [106].

**Figure 7:**
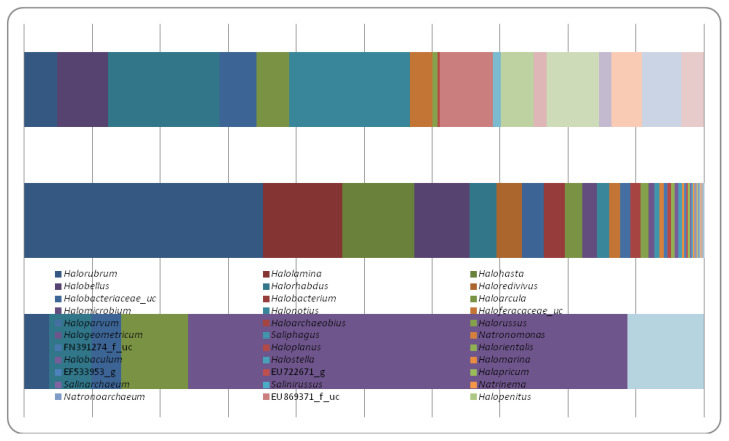
Bar plots showing the genus taxonomic abundance across the samples. Taxa with low abundance (<1%) was eliminated.

At genus level, 37 genera out of 51 currently described [40] were identified in all samples. A clear difference in genera distribution and abundance were observed (Figure 7). With a totally number of 140 OTUs, CDR is considered as the most diverse sample distributed on 43 genera with the abundance of *Halorubrum* genus (35.11%). For CDW sample, with 49 OTUs, 18 genera were identified, with two abundant genera *Halonotius* (17.83%) and *Halorhabdus* (16.28%). Lowest diversity was observed in CDZ sample representing only by 10 OTUs distributed on five genera with the abundance of *Halogeometricum* genus (64.7%). The abundance of such genera in the halite-salt crystal can reflect their physiological and ecological completeness and their survival mechanisms they deploy to cope with the high salt concentrations and anorexic conditions.

Some unclassified genera members (*Halobacteriaceae* Family) were observed in all samples as well. Further comparison showed that *Halorhabdus*, *Halorubrum* and *Haloarcula* genera constitute the main encountered genera within the datasets of the three samples. These results are in accordance with those carried out on several crystals and rock salts samples, where at least one of the archaeal genera has been found [17,43,119].

By comparing the community profiles of CDR and CDW samples collected from the same location (Chott El Jeridi) and characterized by red and white colors respectively, a clear difference on genera composition was observed. CDR sample is characterized by the abundance of *Halorubrum* genus (35.11%), this genus is known by the high capacity in carotenoids biosynthesis [120], which may explain the observed red pigmentation. However, for CDW sample two main abundant genera were observed which *Halotonuis* (17.83%) and *Halorhabdus* (16.28%) are known as light and non-pigmented genus. Indeed, CDZ sample, white halite sample collected from another chott Douz, showed a different profile, with the abundance of *Halogeomtericum genus* (64.70%).

## 5. Haloarchaeal Community Structure between Sites Based on All Approaches

A further comparison of the haloarchaeal genera members found based on the results of culture-independent and dependent methods for each sample revealed a clear distinction in community structures composition between samples, sharing only three classified genera represented by *Halorhabdus*, *Halorubrum*, and *Haloarcula*, and some unclassified *Halobacteriaceae* members (Figure S3). Furthermore, unique genera were observed for each sample citing *Haloferax* and unclassified *Halobacteriaceae* in CDZ; *Halobonum*, *Haladaptatus*, *Haloquadratum*, *Halosimplex,* unclassified euryarchaeote (FN391256) and unclassified *Halobacteriaceae* in CDW; and 30 genera in CDR sample including *Halomarina*, *Haloparvum, Halolamina*, *Halobacterium*, *Halopenitus*, *Halomicrobium*, *Salinirussus*, *Halostella*, *Natronoarchaeum*, *Halorientalis*, *Haloredivivus*, *Haloarchaeobius*, *Saliphagus*, *Halovivax, Halorubellus*, *Salinarchaeum*, *Halapricum*, *Halohasta, Halobaculum*, *Natrinema, Halomicroarcula, Natronomonas, Haloterrigena*, uncultured *Natronomonas*, and unclassified *Halobacteriaceae* members (Table S1).

It is noticeable that, the mainly observed genera, especially those affiliated to *Nanohaloarchaea* family, were only identified with Illumina MiSeq sequencing approach for CDR and CDW samples. Further genera were identified only with the full-length 16S rRNA archaeal libraries approach, the case of *Halonotius*, *Halolamina*, *Haloquadratum*, and *Halorubellus* for CDR sample (Table S1), and *Halonotius* for CDW sample (Table S2). Based on cultured dependent approach, only some isolates affiliated to *Haloferax*, *Halorubrum*, *Halorcula*, *Halogeometricum*, and uncultured haloarchaeal strains have been successfully isolated from all samples which may due to the still unknown growth requirements of the species [121]. The discrepancy between the haloarchaeal diversity analysis achieved by all approaches are in accordance with those reported by other authors [43,122].

## 6. Conclusions

In the present study, we assessed the haloarchaeal diversity in three halite-crystal salts (CDW, CDR, and CDZ) based on culture-independent approaches targeting different regions of 16S rRNA gene sequences including DGGE (V3 regions), 16S rRNA clones libraries (full length gene) and Illumina Miseq sequencing (V3-V4 regions), and classical culture-dependent approach. Results showed unexpected diversity profiles, a clear difference on archaeal profile distribution and abundance between samples based on each approach. The higher diversity was observed with NGS approach, nevertheless, some genera were detected only with 16S rRNA clones’ libraries and cultured approaches. In fact, the amalgamation of all results yielded a comprehensive view of the archaeal diversity represented by members of *Halobacteria* and *Nanohaloarchaea* classes. The main core genera were affiliated to *Halorhabdus*, *Halonotius*, *Halorubrum*, *Haloarcula*, and unclassified members from *Halobacteriacaea* family. Hence, further studies based on whole-genome shotgun (WGS) method could enhance our understanding in the functionality and the mechanisms adopted by haloarchaea members to colonize halite-crystal samples.

## Figures and Tables

**Table 1:** Morphological and molecular characterization of colonies isolated from three halite-crystal samples—namely CDW, CDR, and CDZ

Sampling Site	Origin of Isolation	Strain Code (Acession Number)	Colony Morphology (Number of Colonies Per Morphology)	pH Range	Closest 16S rRNA ^(a)^ (Accession Number)	Similarity
Chott Douz (CDZ)	White salt crust	CDZ60 (MW565884)	Light red and small (1)	6–9	Uncultured *Halorubrum* sp. (FN391189)	99%
		CDZ56 (MW565880)	Orange and big (2)	6–9	*Halorubrum xinjiangense* (AY510707)	99%
		CDZ43 (MW565881)	Light orange and small (2)	6–9	*Halorubrum chaoviator* (AM048786)	99%
Chott DJerid (CDW)	White halite crystal salt	CDW18.2.2 (MW565877)	Mucoid pink and big (3)	6–8	*Haloferax mediterranei* (D11107)	99%
		CDW18.2.3(A) (MW565876)	Red and small (4)	6–9	*Halorubrum chaoviator* (AM048786)	99%
		CDW63 (MW565886)	White and big (4)	6–9	Uncultured haloarchaeon (GQ374937)	99%
		CDW66 (MW565885)	Red (5)	6–9	Uncultured *Halorubrum* sp. (FN391188)	99%
		CDW12 (MW565879)	Light orange (3)	6–9	*Haloarcula vallismortis* (EF645688)	99%
Chott DJerid (CDR)	Pink halite crystal salt	CDR49 (MW565883)	Red small (4)	6–9	Uncultured *Haloarcula* sp. (EF153424)	99%
		CDR50 (MW565887)	Red (4)	5–8	Uncultured haloarchaeon (FN391270)	99%
		CDR52 (MW565878)	Orange and small (3)	6–8	*Halogeometricum borinquense* (AF002984)	99%
		CDR16 (MW565882)	Orange and big (2)	6–9	*Haloarcula vallismortis* (EF645688)	99%
		CDR36 (MW565874)	Red and big (3)	6–8	*Halorubrum chaoviator* (AM048786)	99%
		CDR04 (MW565875)	Small red (4)	6–8	*Halorubrum chaoviator* (AM048786)	98%

^(a)^: all identified genera and species are in italic format.

**Table 2:** Gene sequences (16SrRNA_V3) of related DGGE bands and their closest matches retrieved from RDPII database

Origin of Samples	DGGE Bands (Accession no.)	Closest RDP II Match (Accession no.)_Genus ^(a)^	Similarity Percentage
CDR	5R (MW564201)	*Halorubrum* sp. PJ75-1 (KJ644233)_*Halorubrum*	97.97%
	5 (MW564199)	uncultured archaeon (KT216162)_unclassified_*Halobacteriaceae*	98.17%
	6 (MW564202)	uncultured archaeon (KR920349) unclassified halobacteria	99.57%
CDW	4 (MW564200)	uncultured archaeon (FJ155608)_ unclassified_*Halobacteriaceae*	100%
	3 (MW564198)	uncultured archaeon (EU722680) unclassified_*Halobacteriaceae*	100%
CDZ	1 (MW564203)	*Natronomonas* sp. SA2 (JF950946)_*Natronomonas*	95%
	2 (MW564204)	Uncultured archaeon LC205717_unclassified_*Halobacteriaceae*	95.95%

^(a)^ all identified genera and species are in italic format.

**Table 3:** Operational Taxonomic Units (OTUs) classification and affiliation of 16S rRNA gene clone sequences of CDW, CDR, and CDZ halite-salt crystals samples based on RDP II database

	Clones ID	Closest Relatives (Accession no.)	Similarity (%)	Classification ^(a)^	
	(Accession no. Deposited)				
Sampling Site: CDR					
Total OTUs (0.03 Cut-Off)				Phylum_Family	Genus
**OTU1**	CDR48A (MW565902), CDR49A (MW565903)	Uncultured *Haloquadratum* (FN391222)	98	*Euryarchaeota*_*Halobacteriaceae*	Uncultured *Haloquadratum*
**OTU2**	CDR72 (MW565890)	Uncultured haloarchaeon (GQ375023)	94	*Euryarchaeota*_*Halobacteriaceae*	*Haloquadratum*
**OTU3**	CDR54A (MW565905), CDR67 (MW565893)	Uncultured haloarchaeon (Q375025.1)	99	*Euryarchaeota*_*Halobacteriaceae*	*Haloquadratum*
	CDR42A(MW565897), CDR46A(MW565901), CDR44A (MW565899)	Uncultured Haloquadra (AY987831.1)	98–99	*Euryarchaeota*_*Halobacteriaceae*	*Haloquadratum*
	CDR109A (MW565913)	Uncultured haloarchaeon (GQ375013.1)	99		
**OTU4**	CDR43A (MW565898)	Uncultured haloarchaeon (JN714405.1)	99	*Euryarchaeota*_*Halobacteriaceae*	*Halolamina*
**OTU5**	CDR73A (MW565909)	*Halorubrum chaoviator* (FN391295.1)	99	*Euryarchaeota*_*Halobacteriaceae*	*Halorubrum*
	CDR70 (MW565891), CDR77 (MW565888)	Uncultured haloarchaeon (FN391270.2)	98–99		
**OTU6**	CDR62 (MW567835)	Strain SFH1H061 (FN391295)	95	*Euryarchaeota*_*Halobacteriaceae*	*Halorubrum*
**OTU7**	CDR188 (MW565910)	Uncultured *Halorubrum* (AM947495.1)	99	*Euryarchaeota*_*Halobacteriaceae*	*Halorubrum*
**OTU8**	CDR103A (MW565911)	Uncultured *Halorubrum* (FN391189.1)	98	*Euryarchaeota*_*Halobacteriaceae*	*Halorubrum*
	CDR58A (MW565907)	Uncultured haloarchaeon (FN391244)	99		
**OTU9**	CDR55A (MW565906), CDR53A (MW565904),	Strain SFG1E101 (AM947467)	99	*Euryarchaeota*_*Halobacteriaceae*	*Halonotius*
	CDR68 (MW565892),CDR76A (MW565919)	Uncultured haloarchaeon (GQ374941.1)	99		
**OTU10**	CDR76 (MW565918)	Uncultured haloarchaeon (FN669142.1)	98	*Euryarchaeota*_*Halobacteriaceae*	*Halonotius*
	CDR66 (MW565894), CDR63 (MW565896),	Uncultured haloarchaeon (FN391224)	99		
	CDR82 (MW565920), CDR50A (MW567831)	Uncultured haloarchaeon (FN391226)	98		
**OTU11**	CDR112 (MW565914)	Uncultured haloarchaeon (AM947467)	99	*Euryarchaeota*_*Halobacteriaceae*	*Halonotius*
**OTU12**	CDR64A (MW565908)	Uncultured archaeon (HQ425151.1)	98	*Euryarchaeota*_*Halobacteriaceae*	*Haloarcula*
**OTU13**	CDR75 ((MW565917)	Uncultured *Haloarcula* (EF153424.1)	99	*Euryarchaeota*_*Halobacteriaceae*	*Haloarcula*
	CDR104A (MW565912), CDR74 (MW565916)	Uncultured *Halobacterium* (FN391277.2)	99		
**OTU14**	CDR73 (MW565889), CDR47A (MW567832)	Uncultured *Halobacterium* (FN391286.1)	94	*Euryarchaeota*_*Halobacteriaceae*	*Halorubellus*
**OTU15**		Uncultured haloarchaeon (FN391224.1)	91	*Euryarchaeota*_*Halobacteriaceae*	Unclasssified *halobacteriaceae*
**OTU16**	CDR51A (MW567833), CDR52A (MW567834)	Uncultured *Haloquadratum* (AM947450.1)	95	*Euryarchaeota*_*Halobacteriaceae*	Unclasssified *halobacteriaceae*
**OTU17**	CDR116 (MW565915), CDR45A (MW565900),	Uncultured haloarchaeon (GQ374953)	98–99	*Euryarchaeota*_*Halobacteriaceae*	unclassified_*Halobacteriaceae*
**OTU18**	CDR65 (MW565895)	Uncultured haloarchaeon (GQ375025)	99	*Euryarchaeota*_*Halobacteriaceae*	Unclasssified *halobacteriaceae*
Operational Taxonomic Units (OTUs) classification and affiliation of 16S rRNA gene clone sequences of halite-salt crystals samples based on RDP II database.					
**OTU1**	CDW222 (W563901), CDW208 (MW563889), CDW218 (MW563897), CDW221 (MW563900), CDW209 (MW563890), CDW211 (MW563892), CDW198 (MW563880)	Uncultured *Haloquadratum* sp. (FN994951.1)	98–100	*Euryarchaeota*_*Halobacteriaceae*	*Haloquadratum*
**OTU2**	CDW 182 (MW563870), CDW181 (MW563869)	Uncultured *Haloquadra* sp. (AY987831.1)	94–96	*Euryarchaeota*_*Halobacteriaceae*	*Haloquadratum*
**OTU3**	CDW 184 (MW563871)	Uncultured haloarchaeon clone (GQ374987.1)	96	*Euryarchaeota*_*Halobacteriaceae*	*Haloquadratum*
**OTU4**	CDW195 (MW563877), CDW190 (MW563874)	Uncultured haloarchaeon (FN391230)	99–100	*Euryarchaeota*_*Halobacteriaceae*	Unclassified_*Haloferacaceae*
	CDW197 (MW563879)	Uncultured *Halorubrum* sp. (FN391188.1)	99–100	*Euryarchaeota*_*Halobacteriaceae*	Unclassified_*Haloferacaceae*
**OTU5**	CDW202 MW563884), CDW193 (MW563876)	Uncultured haloarchaeon clone (GQ374985.1)	99	*Euryarchaeota*_*Halobacteriaceae*	*Halorubrum*
**OTU6**	CDW206 (MW567775), CDW194 (MW567774)	Uncultured haloarchaeon clone (AM947463)	91	*Euryarchaeota*_*Halobacteriaceae*	*Halonotius*
**OTU7**	CDW203 (MW563885), CDW199 (MW563881)	Uncultured haloarchaeon clone (AM947467.1)	97–98	*Euryarchaeota*_*Halobacteriaceae*	*Halonotius*
	CDW215 (MW563895), CDW216 (MW563896)	Uncultured haloarchaeon clone (AM947465)	99		
	CDW 219 (MW563898), CDW210 (MW563891)	Uncultured haloarchaeon clone (FN391225.1)	98–99		
	CDW 212 (MW563893), CDW196 (MW563878)	Uncultured haloarchaeon clone (FN391223.1)	99		
	CDW 204 (MW563886)	Uncultured haloarchaeon clone (FN391238.1)	99		
	CDW 207 (MW563888)	Uncultured haloarchaeon clone (FN391224)	99		
**OTU8**	CDW220 (MW563899), CDW205 (MW563887)	Uncultured haloarchaeon clone (FN391270.2)	99	*Euryarchaeota*_*Halobacteriaceae*	*Halorubrum*
	CDW214 (MW563894), CDW200 (MW563882)	Uncultured haloarchaeon clone (FN391295.1)	98		
**OTU9**	CDW224 (MW563902)	Uncultured *Halobacterium* clone (FN391277.2)	97	*Euryarchaeota*_*Halobacteriaceae*	*Haloarcula*
	CDW189 (MW563873), CDW185 (MW563872)	Uncultured archaeon (HQ425180)	99		
**OTU10**	CDW228 (MW563905)	Uncultured haloarchaeon clone (JN714408.1)	95	*Euryarchaeota*_*Halobacteriaceae*	Unclassified_*Halobacteriaceae*
	CDW229 (MW563906)	Uncultured *Halobacterium* clone (FN391290.1)	98		
**OTU11**	CDW192 (MW563875), CDW226 (MW563903)	Uncultured archaeon clone (EU722673.1)|	95–98	*Euryarchaeota*_*Halobacteriaceae*	Unclassified_*Halobacteriaceae*
**OTU12**	CDW227 (MW563904), CDW201 ( MW563883)	Uncultured *Halobacterium* sp. (AM947502)	98–99	*Euryarchaeota*_*Halobacteriaceae*	*Halorubrum*
	CDW197 (MW563879)	Uncultured *Halobacterium* sp. (FN391188.1)	99		
Operational Taxonomic Units (OTUs) classification and affiliation of 16S rRNA gene clone sequences of halite-salt crystals samples based on RDP II database.					
**OTU1**	CDZ122 (MW564064), CDZ149 ((MW564056), CDZ141 (MW564061), CDZ127 (MW564063), CDZ138 (MW564062), CDZ143 (MW564059)	Uncultured archaeon (FJ172053)	99	*Euryarchaeota*_*Halobacteriaceae*	*Halorubrum*
	CDZ144 (MW564058)	Uncultured haloarchaeon (EF533955.1)	99	*Euryarchaeota*_*Halobacteriaceae*	
	CDZ154 (MW564054)	Uncultured *Halobacterium* (FN994971.1)	99	*Euryarchaeota*_*Halobacteriaceae*	
**OTU2**	CDZ142 (MW564060), CDZ148 (MW564057), CDZ150 (MW564055)	Uncultured archaeon (FJ172058)	99	*Euryarchaeota*_*Halobacteriaceae*	*Halorubrum*

^(a)^ all identified family, genera and species are in italic format.

**Table 4:** Alpha diversity of archaeal community of different halite-crystal salt samples (CDR, CDW, CDZ) of 16S rRNA Clones libraries and metataxonomic approaches

	Alpha Diversity				
Samples and Approaches	Total of Cloned Sequences/Valid Reads	Total OTU0.03 ^a^	Shannon Index	Chao Index	Good’s Coverage
**16S rRNA Clones libraries approach**					
**CDR**	38	18	2.67	2.0	76.31
**CDW**	40	12	2.25	12.16	95
**CDZ**	11	0.2	0.58	25.2	100
**Amplicon analysis**					
**CDZ**	731	10	1.245	11	99.73
**CDW**	2119	49	3.255	49.4	99.9
**CDR**	3198	131	3.559	134.7	99.7

^a^: Cut-off value of 3% was used for statistical analysis.

## Data Availability

All 16S rRNA sequences identified in this study were deposited in GenBank with the following accession numbers: MW565874-MW565887 for isolates; MW564198-MW564204 for DGGE; MW564054-MW564064 for CDZ sample; MW563869-MW563906 for CDW; and MW565874-MW565920 for CDR sample.

## Supplementary Materials

The following are available online at https://www.mdpi.com/article/10.3390/biology10050397/s1, Figure S1: Archaeal DGGE patterns of 16S rDNA gene sequences of the V3 variables regions’ (567 bp) obtained from different salt samples. Lane 1: CDR; lane 2: CDW; lane 3: CDZ. Bands marked with a numbered asterisk were excised from gel, re-amplified and sequenced. The gradient of the urea and formamide ranged from 20% to 55%, Figure S2: Genus-level rarefaction curves of CDZ, CDR and CDW sample based on the 16S rDNA sequences analyzed, Figure S3: Venn diagrams showing the unique and shared OTUs (3% distance level) identified based on cultured, 16S rRNA libraries, DGGE and metataxonomic approaches, for CDR, CDW and CDZ respectively, Table S1: Unique and shared genera between CDW, CDR and CDZ samples based on cultured, 16S rRNA libraries, DGGE and metataxonomic approaches, Table S2: List of shared and unique genera identified in CDW sample based on both cultured and uncultured-dependant approaches
