# Supplementary material for: Assessment of 16S rRNA Gene-Based Phylogenetic Diversity of Archaeal Communities in Halite-Crystal Salts Processed from Natural Saharan Saline Systems of Southern Tunisia
Source: Biology (Basel). 2021 May 4;10(5):397. doi: 10.3390/biology10050397 (PMC8147861; doi:10.3390/biology10050397)
Supplement: Supplementary file 1 [file biology-10-00397-s001.zip › biology-1143910-SI.pdf]

Assessment of 16S rRNA Gene-based Phylogenetic Diversity of Archaeal Communities in Halite-crystals Salts Processed from Natural Saharan Saline Systems of Southern Tunisia

Afef Najjari[1], Panagiota Stathopoulou[2], khaled Elmnasri[3], Faten Hasnaoui[1], Ines Zidi[1], Haitham Sghaier[3,4], Hadda I Ouzari[1], Ameur Cherif[3] and George Tsiamis[2]

[1]Université Tunis El Manar, Faculté des Sciences de Tunis, LR03ES03 Laboratoire de Microbiologie et Biomolécules Actives, 2092, Tunis, Tunisi

[2] Laboratory of Systems Microbiology and Applied Genomics, Department of Environmental Engineering, University of Patras, 2 Seferi Str., 30100 Agrinio, Greece

[3]Univ. Manouba, ISBST, LR11-ES31 BVBGR, Biotechpole Sidi Thabet, 2020, Ariana, Tunisia.

[4]Laboratory "Energy and Matter for Development of Nuclear Sciences" (LR16CNSTN02), National Center for Nuclear Sciences and Technology (CNSTN)

Correspondence: Afef Najjari, Université Tunis El Manar, Faculté des Sciences de Tunis, LR03ES03 Laboratoire de Microbiologie et Biomolécules Actives, 2092, Tunis, Tunisie. email: afef.najjari@fst.utm.tn *

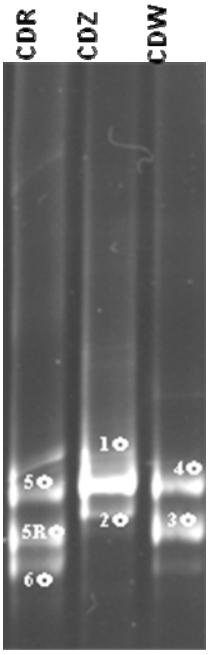

Figure S1. Archaeal DGGE patterns of 16S rDNA gene sequences of the V3 variables regions' (567 bp) obtained from different salt samples. Lane1: CDR; lane2: CDW; lane3: CDZ. Bands marked with a numbered asterisk were excised from gel, re-amplified and sequenced. The gradient of the urea and formamide ranged from 20% to 55%.

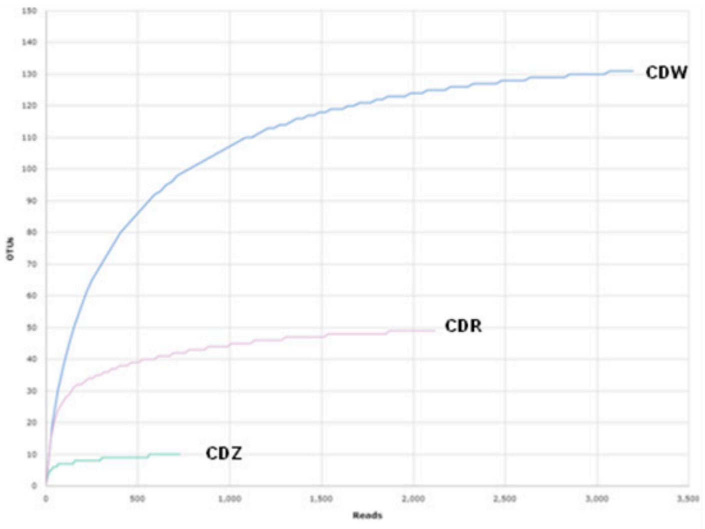

Figure S2: Genus-level rarefaction curves of CDZ, CDR and CDW sample based on the 16S rDNA sequences analyzed.

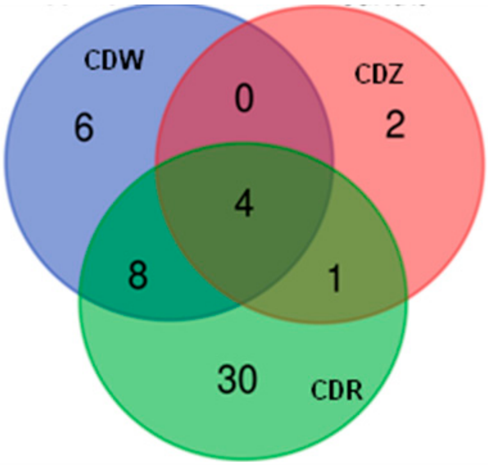

Figure 3. Venn diagrams showing the unique and shared OTUs (3% distance level) identified based on cultured, 16S rRNA libraries, DGGE and metataxonomic approaches, for CDR, CDW and CDZ respectively.

Table S1: Unique and shared genera between CDW, CDR and CDZ samples based on cultured, 16S rRNA libraries , DGGE and metataxonomic approaches

Samples	Total	Genera
CDR/CDW/CDZ	4	*Halorhabdus*, *Halorubrum*, *Haloarcula*, unclassified *Halobacteriaceae*
CDW/CDZ	8	*Halonotius*, *Halorussus*, *Halovenus*, *Haloplanus,* Uncultured haloarchaeon (JN714452), unclassified *Haloferacaceae*, Uncultured haloarchaeon (EU869371), *Halobellus*
CDZ/CDZ	1	*Halogeometricum*
CDW	6	*Halobonum*, uncultured euryarchaeote (FN391256), Haladaptatus, Haloquadratum, Unclassified in higher taxonomic rank, *Halosimplex*
CDZ	2	*Haloferax*, unclassified *Halobacteriaceae*
CDR	30	uncultured *Natronomonas* (FN391194), *Halomarina*, *Haloparvum, Halolamina*, Uncultured haloarchaeon (EU869371), *Halobacterium, Halopenitus, Halomicrobium, Salinirussus, Halostella, Natronoarchaeum, Halorientalis,* uncultured archaeon (EU722671), *Haloredivivus, Haloarchaeobius, Saliphagus, Halovivax ,Halorubellus, Salinarchaeum, Halapricum, Halohasta, Halobaculum*, uncultured archaeon (KI543180), *Natrinema*, Uncultured haloarchaeon (FN391274), *Halomicroarcula*, *Natronomonas*, *Haloterrigena*, unclassified *Natrialbaceae*, Uncultured haloarchaeon (EF533953)

Tables S2: List of shared and unique genera identified in CDW sample based on both cultured and uncultured-dependant approaches

Approches	Total	Shared and unique genera
16S rRNA libraries/Cultured /DGGE /Miseq analysis	2	*Halorubrum*, *Haloarcula*
16S rRNA libraries/ Miseq analysis	1	*Haloquadratum*
16S rRNA libraries	2	Unclassified *Halobacteriaceae*, *Halonotius*
Cultured	3	Strain ZA20911, *Haloferax*, Strain LDS1
DGGE	1	unclassified_*Halobacteriaceae*
Miseq analysis	14	*Halobonum Haloplanus Halovenus Unclassified phylum Halorhabdus unclassified halobacteriacaea* (EU869371) *Haladaptatus unclassified halobacteriacaea* (JN714452) *unclassified halobacteriacaea* (FN391256) *Haloferacaceae_uc unclassified Halobacteriaceae Halorussus Halosimplex Halobellus*

Tables S3: List of shared and unique genera identified in CDR sample based on both cultured and uncultured-dependant approaches

Approches	Total	Shared and unique genera
16S rRNA libraries/Cultured / Miseq analysis	1	*Halorubrum*
/ DGGE / Miseq analysis	1	*Natronomonas*
Cultured	2	Strain 209ZB09 (FN391189), unclassified_*Halobacteriaceae*
DGGE	1	unclassified_*Halobacteriaceae* (LC205717)
Miseq analysis	40	*Halomarina, Haloparvm, Halolamina, Halobacterium, Unclassified Halobacteriaceae (EF533953) , Halorhabdus,Halonotius, Halopenitus, Halomicrobium, Salinirussus ,Halostella, Natronoarchaeum Halorientalis Unclassified Natrialbaceae Haloredivivus Halogeometricum Halorussus Haloarchaeobius Unclassified Halobacteriaceae Unclassified Haloferacaceae (KI543180) Saliphagus Halovivax Unclassified Haloferacaceae (JN714452) Halorubellus Unclassified Nanosalina (FN391274) Unclassified Nanosalina (EU869371) Halovenus Haloplanus Halapricum Salinarchaeum Halohasta Unclassified Haloferacaceae Halobaculum Natrinema Halomicroarcula Unclassified Halobacteriaceae (EU722671) Unclassified Haloferacaceae (FN391194) Haloterrigena Haloarcula Halobellus*

Table S4: List of shared and unique genera identified in CDZ sample based on both cultured and uncultured-dependant approaches

Approches	Total	Shared and unique genera
16S rRNA libraries/Cultured /DGGE / Miseq analysis	1	*Halorubrum*
16S rRNA libraries /Cultured/ Miseq analysis	1	*Haloarcula*
Cultured/ Miseq analysis	1	*Halogeometricum*
16S rRNA libraries	4	*Halonotius, Halolamina*, *Haloquadratum, Halorubellus*
Cultured	2	Strain SFH1A041, Strain ntu 13
Miseq analysis	5	*Haloferax, Halobacteriaceae*_uc, *Halorhabdus, Haloarcula, Halogeometricum, Halorubrum*
